# The microbiology of deep-sea hydrothermal vent plumes: ecological and biogeographic linkages to seafloor and water column habitats

**DOI:** 10.3389/fmicb.2013.00124

**Published:** 2013-05-21

**Authors:** Gregory J. Dick, Karthik Anantharaman, Brett J. Baker, Meng Li, Daniel C. Reed, Cody S. Sheik

**Affiliations:** ^1^Department of Earth and Environmental Sciences, University of MichiganAnn Arbor, MI, USA; ^2^Department of Ecology and Evolutionary Biology, University of MichiganAnn Arbor, MI, USA; ^3^Center for Computational Medicine and Bioinformatics, University of MichiganAnn Arbor, MI, USA

**Keywords:** chemosynthesis, chemoautotroph, deep-sea, biogeochemistry, hydrothermal, vent, biogeography

## Abstract

Hydrothermal plumes are an important yet understudied component of deep-sea vent microbial ecosystems. The significance of plume microbial processes can be appreciated from three perspectives: (1) mediation of plume biogeochemistry, (2) dispersal of seafloor hydrothermal vent microbes between vents sites, (3) as natural laboratories for understanding the ecology, physiology, and function of microbial groups that are distributed throughout the pelagic deep sea. Plume microbiology has been largely neglected in recent years, especially relative to the extensive research conducted on seafloor and subseafloor systems. Rapidly advancing technologies for investigating microbial communities provide new motivation and opportunities to characterize this important microbial habitat. Here we briefly highlight microbial contributions to plume and broader ocean (bio)geochemistry and review recent work to illustrate the ecological and biogeographic linkages between plumes, seafloor vent habitats, and other marine habitats such as oxygen minimum zones (OMZs), cold seeps, and oil spills. 16S rRNA gene surveys and metagenomic/-transcriptomic data from plumes point to dominant microbial populations, genes, and functions that are also operative in OMZs (SUP05, ammonia-oxidizing Archaea, and SAR324 *Deltaproteobacteria*) and hydrocarbon-rich environments (methanotrophs). Plume microbial communities are distinct from those on the seafloor or in the subsurface but contain some signatures of these habitats, consistent with the notion that plumes are potential vectors for dispersal of microorganisms between seafloor vent sites. Finally, we put forward three pressing questions for the future of deep-sea hydrothermal plume research and consider interactions between vents and oceans on global scales.

## INTRODUCTION

Deep-sea hydrothermal plumes occur where seafloor vents inject hydrothermal fluids replete with potential microbial energy sources such as H_2_S, Fe, Mn, CH_4_, and H_2_ into the deep oceans. These hot, chemically reduced fluids rapidly mix with cold, oxidizing seawater, forming hydrothermal plumes that rise hundreds of meters off the seafloor and disperse hundreds of kilometers away from their source. Because of their extensive spatial coverage and easily detectable hydrothermal signals (Fe, Mn, turbidity, Helium-3), plumes played an important role in the history of deep-sea hydrothermal vent research (Lupton and Craig, 1981) and continue to be utilized for discovery of new seafloor hydrothermal systems (German et al., 2010). Hydrothermal plumes are highly variable in terms of scale and chemical and physical properties, and can be detected by a variety of methods (chemical, physical, optical), thus the definition of a plume depends on the parameter being measured (Lupton, 1995). For many of the geochemical and microbiological processes of interest here, “plume” often refers to hydrothermal fluid that has been heavily diluted by seawater (e.g., ~1:10,000), but some work has addressed microbial processes in the rising portion of the plume where hydrothermal constituents are more concentrated.

**FIGURE 1:**
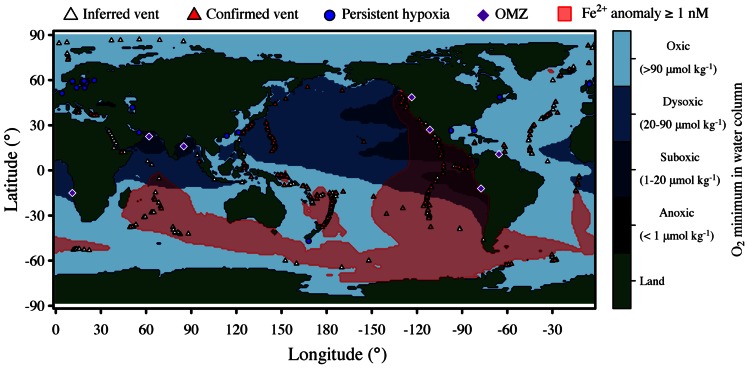
**Global distribution of deep-sea hydrothermal systems, iron anomalies due to hydrothermal inputs, and low oxygen environments**. Blue shading indicates minimum concentration of O_2_ in the water column with intervals defined by Wright et al. (2012). Iron data is from Tagliabue et al. (2010). Major oxygen minimum zones (OMZ) and sites of persistent hypoxia [year-round or near year-round (Diaz and Rosenberg, 2008)] are also indicated.

Hydrothermal plumes are found at vents sites distributed globally along the mid-ocean ridge system (**Figure 1**) at a frequency correlated to seafloor spreading rate (Beaulieu et al., 2012). Deep-sea vent systems continue to be discovered at a rapid pace; over 500 vent fields are now known, nearly double the number known before the year 2000 (Beaulieu et al., 2012). Yet much of the mid-ocean ridge system remains unexplored, especially at ultra-slow spreading ridges, which have only recently been recognized to host hydrothermal activity (German et al., 2010) and are particularly abundant in the Arctic and Southern oceans. Hydrothermal venting in shallow waters is also widespread (Prol-Ledesma et al., 2005), but here we focus only on deep-sea systems. Given the global distribution and extent of hydrothermal venting, it is clear that deep-sea vents exert significant influence on the chemistry of the global oceans (Elderfield and Schultz, 1996). A recent modeling study of hydrothermal contributions to the marine iron inventory (Tagliabue et al., 2010) highlights the global impacts of vents (**Figure 1**).

## MICROBIAL MEDIATION OF PLUME BIOGEOCHEMISTRY: TRACE ELEMENTS, PHOSPHORUS AND CARBON

The impact of deep-sea hydrothermal vents on ocean chemistry involves several processes that are influenced by microbial activities in hydrothermal plumes. First, vents are thought to be a significant source of Fe and Mn to the oceans because their concentrations in hydrothermal fluids are up to 10^6^ times that of background seawater (Elderfield and Schultz, 1996; Tagliabue et al., 2010; Sander and Koschinsky, 2011). The oceanic fate of these metals is influenced by scavenging and oxidation, which are promoted by microorganisms (Cowen and Bruland, 1985; Cowen et al., 1986; Mandernack and Tebo, 1993; Dick et al., 2009), and by binding with organic matter, which is presumably derived from microbial activity (Bennett et al., 2008; Toner et al., 2009; Breier et al., 2012; Holden et al., 2012). Second, the iron and manganese oxides produced by microbial oxidation are extraordinarily reactive (Goldberg, 1954; Tebo et al., 2004) and thus remove phosphorous and trace elements (rare earth elements, potassium, vanadium, arsenic, chromium, uranium) from seawater via scavenging and co-precipitation reactions (Feely et al., 1998; German and Von Damm, 2004). Because of the rapid mixing of seawater with hydrothermal fluids and the large volumes of plumes, the entire volume of the global oceans cycles through hydrothermal plumes and is scavenged of reactive elements on relatively short times scales (2.4 × 10^5^ y; Kadko, 1993). Thus plumes essentially act as a filter for the global oceans, scavenging them of phosphorous, rare earth elements, and trace metals, and acting as a chemical sink for these elements (Kadko, 1993). As these Fe and Mn oxides and their scavenged elements are deposited to the seafloor, they form metalliferous sediments that potentially preserve a record of seawater nutrient status and chemistry that is valuable from paleoceanographic perspectives (Feely et al., 1998). Similarly, banded iron formations, which are likely sourced from hydrothermal activity, provide a Precambrian record of ocean chemistry (Konhauser et al., 2009; Planavsky et al., 2011). Thus, microbially mediated metal oxide formation and the properties of the resulting biogenic minerals in plumes influence scavenging reactions and outcomes in terms of ocean chemistry, and understanding these processes is critical for interpretation of the sedimentary record for paleoceanographic purposes.

The third process by which plume microorganisms mediate broader ocean biogeochemistry is chemosynthetic fixation of carbon. Chemosynthetic activity at vents was recognized upon the initial discovery of deep-sea hydrothermal vent ecosystems (Jannasch and Wirsen, 1979) and in early plume studies (Winn et al., 1986) yet the magnitude of chemosynthesis in plumes remains poorly constrained. Based on extrapolation of data from the Southern East Pacific Rise to the global oceans, Maruyama et al. (1998) estimated that net primary production in plumes represents 0.1–1% of total marine photosynthetic net primary production. Because only a small fraction of surface organic carbon reaches the deep oceans, the hydrothermal contribution could represent up to 25% of the global deep ocean organic carbon inventory (Maruyama et al., 1998). Thermodynamic models support the idea that plumes are a significant source of chemosynthetically derived organic carbon to the deep oceans (McCollum, 2000), and several observational studies confirm an important contribution of plumes to deep-sea organic carbon on regional scales (De Angelis et al., 1993; Cowen and German, 2003; Lam et al., 2004, Lam et al., 2008). Indeed, plumes are enriched with organic carbon, some of which is labile, and are responsible for dispersing it kilometers away from vent sites (Roth and Dymond, 1989; Cowen et al., 2001; Shackelford and Cowen, 2006; Lam et al., 2008; Bennett et al., 2011a,b). More recently, transcriptomic evidence confirms that autotrophy is a prevalent process in plumes (Baker et al., 2012; Lesniewski et al., 2012; Anantharaman et al., 2013).

Two recent developments provide fresh motivation for re-examining the global impact of hydrothermal vents on the chemistry and biology of the oceans. First, hydrothermal activity along the mid-ocean ridges is more common than previously recognized, especially at slow-spreading systems (German et al., 2010; Beaulieu et al., 2012). Slow-spreading ridges represent a large but poorly explored portion of the global mid-ocean system and host high-energy, H_2_-rich systems that are potential hotspots for chemosynthesis (Amend et al., 2011). Second, the discovery of cryptic biogeochemical cycling of sulfur (Canfield et al., 2010) and widespread chemolithoautotrophy in the broader pelagic oceans (Aristegui et al., 2009; Swan et al., 2011) suggests that current models underestimate chemosynthesis and raise questions regarding ecological connections between plume and other pelagic environments, which we consider below.

## MICROBIAL COMMUNITIES IN DEEP-SEA HYDROTHERMAL PLUMES

**FIGURE 2:**
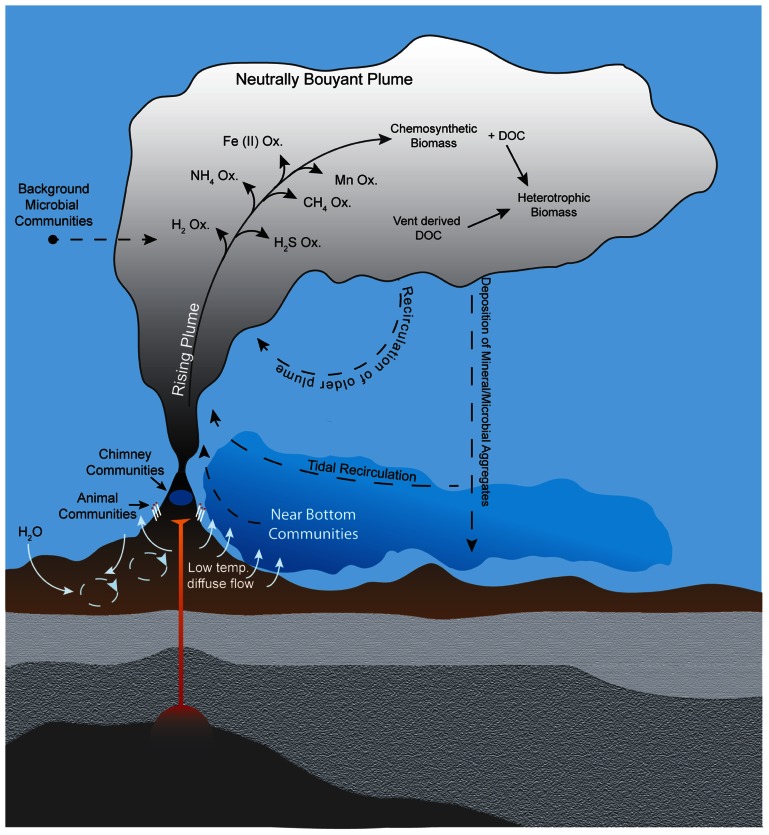
**Potential sources of plume microorganisms include microbial communities in background seawater, vent chimneys, near-vent animal symbioses, subsurface environments, near-bottom waters, and recirculation of aged plumes.** Microbial growth within the plume also shapes the plume community, including utilization of hydrothermally sourced electron donors for chemosynthesis as well as heterotrophic consumption of organic carbon produced chemosynthetically or hydrothermally. Hydrothermal plumes contain distinct regions (e.g., the rising plume and neutrally buoyant plume) with steep gradients of physical and chemical properties that likely hold distinct microbial communities.

Despite the well-recognized importance of microorganisms in the biogeochemistry of hydrothermal plumes, few studies have characterized microbial communities that inhabit them. Thus the physical source, taxonomic composition, and ecological nature of these organisms remain poorly understood. Potential sources of microbes for deep-sea hydrothermal plumes can be divided into three broad categories: (i) seafloor (or sub-seafloor) communities, (ii) background deep seawater communities, or (iii) growth *within* the plume (**Figure 2**). The traditional view is that plume microbes are likely sourced from highly productive biological communities that inhabit vent chimneys and surrounding areas (Winn et al., 1986). Such sources could also include bottom water that is heavily influenced by low-temperature diffuse flow (Kadko et al., 1990), which may be responsible for geochemical flux comparable to focused hydrothermal venting and contains microbes from the subsurface biosphere (Wankel et al., 2011; Akerman et al., submitted). Indeed, tracer studies indicate that diffuse flow and larvae of vent fauna can be entrained into plumes (Jackson et al., 2010). Alternatively, plume microbes could be derived primarily from ambient background seawater, which seems feasible given that plumes are a mix of >99% seawater and ~0.01% hydrothermal fluid (Lupton et al., 1985) and that benthic and pelagic habitats differ greatly (Zinger et al., 2011). Regardless of the original source of microbes, it is likely that plume communities are dynamic in time and space. Shifts in microbial community structure are to be expected as the geochemical environment of hydrothermal fluids evolves with plume age (i.e., become more dilute and oxidized). Consistent with this notion are observations of progressive removal of electron donors (Kadko et al., 1990) and morphological evidence of changes in microbial communities with plumes age (Cowen and Li, 1991). However, only recently has plume microbial diversity been sampled and analyzed with molecular tools in a spatially resolved manner.

Many studies have reported elevated microbial biomass and activity in plumes relative to background, suggesting that plume microbes are distinct from those in the ambient water column (Winn et al., 1986; Naganuma et al., 1989; Juniper et al., 1998; Maruyama et al., 1998; O’Brien et al., 1998; Lam et al., 2004, Lam et al., 2008; Dick et al., 2009). In one of the first applications of molecular tools to deep-sea hydrothermal plumes, Sunamura et al. (2004) showed that the Suiyo Seamount hydrothermal plume is dominated by just two phylotypes, one group of *Gammaproteobacteria *and one group of *Epsilonproteobacteria*. Interestingly, both of these phylotypes were most closely related (at the time) to symbionts of hydrothermal vent animals – the *Gammaproteobacteria* to bivalve gill symbionts and the *Epsilonproteobacteria* to ectosymbionts of the tubeworm *Riftia pachyptila* and shrimp *Rimicaris exoculata* (Sunamura et al., 2004). The *Gammaproteobacteria*, designated “SUP05,” were also found to dominate low-temperature diffuse flow emanating from a bivalve-colonized mound (99% of cells; Sunamura et al., 2004), raising the possibility that microbial communities in the subsurface or animal symbioses are sources of plume microbes (see further discussion of links between plumes and symbionts below). Indeed, SUP05-like sequences have also been retrieved from diffuse hydrothermal fluids on the seafloor at the Juan de Fuca ridge (Huber et al., 2003; Bourbonnais et al., 2012; Anderson et al., 2013). However, recent rRNA gene surveys show that SUP05 are also widely distributed in pelagic environments, raising the question of whether the connection between plumes and subsurface is physical (i.e., transport only) or ecological (i.e., actively operating in similar niches in both environments) in nature. An extreme example of the physical transport of seafloor and/or subsurface material to the water column is “snowblower” vents that discharge elemental sulfur and bacterial filaments (Haymon et al., 1993; Crowell et al., 2008). Thermophilic microbes derived from the subsurface have also been observed in eruptive event plumes (Summit and Baross, 1998). *Epsilonproteobacteria* are also commonly encountered in vent seafloor environments (Nakagawa et al., 2005; Campbell et al., 2006; Zinger et al., 2011), again highlighting potential connections between the seafloor and plumes. Clear signals of *Epsilonproteobacteria* and other seafloor hydrothermal microbes have been observed in plumes at the Mid-Cayman Rise (German et al., 2010), in the Iheya hydrothermal field (Nakagawa et al., 2005), the Logatchev hydrothermal plume (Perner et al., 2013), and in descending particles from plumes at the East Pacific Rise, which are genetically distinct from surrounding seawater (Sylvan et al., 2012). Recent studies employing fine-scale phylogenetic approaches and coupled DNA and RNA approaches hold great promise for elucidating the niche space and distribution of sulfur-oxidizing *Gammaproteobacteria* and *Epsilonproteobacteria* in subsurface, seafloor, plume, and background environments. Anderson et al. (2013) noted partitioning of distinct clades of sulfur-oxidizing *Gammaproteobacteria* in vent environments (SUP05 in plumes and animal symbioses) versus others [Arctic96BD-19 in background and oxygen minimum zones (OMZs)] and noted that sulfide concentration likely controls the balance of *Gammaproteobacteria* versus *Epsilonproteobacteria*. Consistent with that view, coupled RNA and DNA analyses revealed showed that *Epsilonproteobacteria* are more active in the reducing environment of the subseafloor, whereas *Gammaproteobacteria* are more active at the seafloor where mixing with seawater is more prevalent (Akerman et al., submitted).

In contrast with the hypothesis that seafloor environments are the major source of plume biota, microbial communities in hydrothermal plumes at Guaymas Basin in the Gulf of California are distinct from those of the underlying seafloor habitats such as hydrothermal sediments or chimneys (Dick and Tebo, 2010). Rather, Guaymas Basin plume communities closely resemble those from background seawater samples taken just above the plume or in the neighboring Carmen Basin, which is 100 km away and does not host hydrothermal activity. Metagenomic and metatranscriptomic data further reinforces the plume-water column connection, showing that the metabolically active microbes in Guaymas plumes are pelagic rather than benthic in nature, and suggesting an ecological boundary between seafloor and plume (Lesniewski et al., 2012). These studies interpreted the above-plume and Carmen Basin samples as true background communities, and this is supported by the absence of detectable physical and chemical tracers of hydrothermal activity in those samples. However, another possibility is that this deep seawater surrounding Guaymas Basin is impacted by microbes that are exported from the highly productive chemoautotrophic plumes. Processes that could facilitate export include ascending and descending particles and migratory zooplankton (Cowen et al., 2001), buoyant transparent exopolymeric substances (Shackelford and Cowen, 2006; Prieto and Cowen, 2007), and large scale advection such as mesoscale eddies (Adams et al., 2011). Regional influence of vents on deep-sea microbial communities may be particularly important in the Gulf of California, where restricted basins could limit dispersal of plumes and mixing with true non-hydrothermally-impacted seawater. We will re-visit the potential impact of hydrothermal plumes on the broader deep oceans below.

## ECOLOGICAL AND BIOGEOGRAPHIC LINKAGES BETWEEN HYDROTHERMAL PLUMES AND OTHER MARINE HABITATS

**FIGURE 3:**
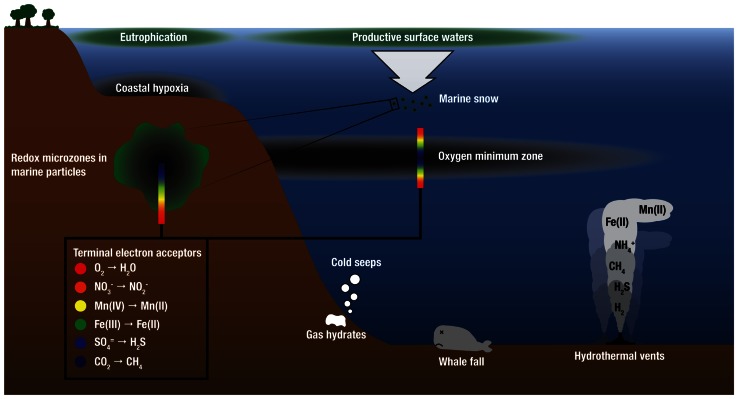
**Schematic water column profile showing selected marine habitats that are sources of electron donors for chemosynthetic growth and support microbial communities similar to those found in deep-sea hydrothermal plumes**. Electron donors for microbial growth are supplied by geothermal sources at deep-sea vents and as the products of anaerobic microbial respiration in low-O_2_ environments. OMZ and redox schematics after Wright et al. (2012).

There is growing recognition that chemical species that fuel microbial growth in hydrothermal plumes (H_2_, various sulfur species, ammonium, and iron) also support microbial growth in marine environments well beyond hydrothermal systems, including OMZs, oils spills, and cold seeps (Paull et al., 1984; Lösekann et al., 2007; Tavormina et al., 2008, 2010; Redmond et al., 2010), whale falls (Baco and Smith, 2003; Tringe et al., 2005; Goffredi and Orphan, 2010), and within microenvironments of organic-rich particles in the oxic water column (Karl et al., 1984; **Figure 3**). Indeed, recent molecular surveys show that microorganisms that are abundant in deep-sea hydrothermal plumes are also abundant in these other marine habitats. SUP05 and another group of uncultivated putative sulfur-oxidizing bacteria, SAR324 *Deltaproteobacteria*, are abundant in OMZs, where they play important roles in linking the sulfur and nitrogen cycles (Lavik et al., 2009; Walsh et al., 2009; Canfield et al., 2010; Stewart et al., 2012; Wright et al., 2012). These two groups have also been identified in the dark pelagic oceans (e.g., Swan et al., 2011; Ghiglione et al., 2012). In addition, methanotrophic populations and functional genes for methane oxidation (particulate methane monooxygenase; *pMMO*) that are prevalent in the Guaymas Basin hydrothermal plume are closely related to those in plumes of the Deepwater Horizon oil spill (Lesniewski et al., 2012; Li et al., unpublished). Cultures also support connections between plumes and other marine environments; close relatives of the Mn(II)-oxidizing alphaproteobacterium SI85-9A1 (>99% 16S rRNA gene sequence identity), which was originally isolated from the Saanich Inlet oxic/anoxic interface (Dick et al., 2008), have been isolated from the surface of *Alvinella pompejana* tubeworms at 9°N East Pacific Rise (Anderson et al., 2009), and from plumes of the Lau Basin. Finally, *Halomonas* and *Marinobacter* species detected in hydrothermal plumes are present throughout the oceans (Kaye and Baross, 2000, 2004; Kaye et al., 2011). Hence, plume ecological niches and the microorganisms that fill them appear to be widespread in the oceans, and understanding their distribution is paramount for understanding the dispersal of plume microorganisms, the “inoculation” of plumes with microbes from background seawater, and ultimately for understanding the distribution and abundance of chemoautotrophy throughout the global oceans. Below we focus on comparisons of plumes to OMZs and seafloor environments.

### CONNECTIONS TO OXYGEN MINIMUM ZONES

Oxygen minimum zones are widely distributed in the oceans and are expanding due to anthropogenic global change (Wyrtki, 1962; Stramma et al., 2008; Wright et al., 2012). Reduced O_2_ concentrations favor alternative terminal electron acceptors, the products of which drive chemoautotrophic metabolisms. Hence, microbial metabolisms that take advantage of redox gradients mediate OMZ biogeochemistry and contribute significantly to the global cycling of nitrogen and greenhouse gasses (Wright et al., 2012). OMZs in which the concentration of O_2_ falls below 20 μM, a threshold below which some anaerobic metabolisms operate (Kalvelage et al., 2011; Ulloa et al., 2012), overlap geographically with the mid-ocean ridge system, especially in the Eastern Pacific (**Figure 1**). Although OMZs and deep-sea hydrothermal vent plumes are typically separated vertically by 1500 m or more, communication between these habitats likely occurs via sinking particles. Whereas nitrogen cycling was classically thought to dominate the microbial ecology and metabolism of OMZs, cryptic sulfur cycling has also been recently demonstrated in the Eastern Tropical South Pacific OMZ (Canfield et al., 2010). Such cryptic sulfur cycling occurs when sulfide produced by sulfate reduction is rapidly oxidized back to sulfate. Though easily overlooked by chemical methods, numerous reports of abundant sulfur-oxidizing autotrophs in OMZs (Stevens and Ulloa, 2008; Lavik et al., 2009; Walsh et al., 2009) and even in oxic waters (Swan et al., 2011) suggest that this process may be widespread in the broader oceans.

**FIGURE 4:**
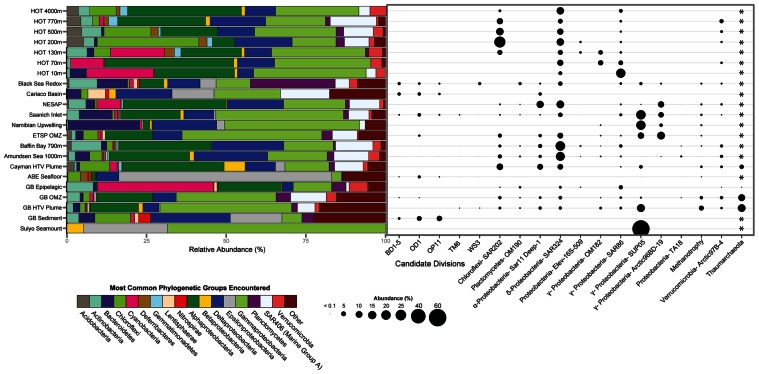
**Microbial community structure and abundance of key populations in plumes and other marine habitats.** 16S rRNA gene clone and pyrosequencing reads were downloaded from Genbank or VAMPS (vamps.mbl.edu) repositories and classified to the Silva v. 111 database (Quast et al., 2013) using the Mothur software package (Schloss et al., 2009). Sequences were taken from: Hawaii Ocean Time Series (HOT; Pham et al., 2008), Black Sea (Fuchsman et al., 2011), Cariaco Basin (Madrid et al., 2001), Northeast Subarctic Pacific (Wright et al., 2012), Saanich Inlet (Walsh et al., 2009), Namibian upwelling zone (Lavik et al., 2009), Eastern Tropical South Pacific OMZ (ETSP; Stevens and Ulloa, 2008), Baffin Bay and Amundsen Sea (Ghiglione et al., 2012), Mid-Cayman Rise hydrothermal vent plume (German et al., 2010), ABE hydrothermal vent seafloor at Lau Basin (Flores et al., 2012), Guaymas Basin (Anantharaman et al., 2013), Guaymas Basin Sediment (Teske et al., 2002), and Suiyo Seamount (Sunamura et al., 2004). *Primer sets used were bacterial specific and did not allow for comparison of Archaea.

To evaluate potential connections between the microbial ecology of deep-sea hydrothermal plumes and other marine environments, we compared the most abundant microbial groups across a variety of pelagic and benthic habitats including both oxic and anoxic as well as hydrothermal and non-hydrothermal environments (**Figure 4**). Although there are obviously a variety of environmental and biogeographic factors that shape microbial community structure in these diverse habitats, there are some striking similarities in terms of the organisms that dominate. SUP05 are among the most abundant microbial groups in the Guaymas Basin and Suiyo Seamount hydrothermal plumes, Saanich Inlet and ETSP OMZs, and in coastal waters of the Benguela upwelling zone off Namibia (Lavik et al., 2009). SUP05 are also found at appreciable abundance in the OMZ of Guaymas Basin (Anantharaman et al., 2013) and in the Black Sea redoxcline (Fuchsman et al., 2011). The SAR324 group of *Deltaproteobacteria* (also known as the Marine Group B), which has recently been implicated in hydrocarbon and sulfur metabolism and autotrophy (Swan et al., 2011; Li et al., unpublished; Sheik et al., unpublished), is also abundant in widespread environments (**Figure 4**). It should be noted there is considerable diversity within these important groups, which likely reflects specific ecological adaptations. For example, there are clades of SAR324 that appear to be specific to Saanich Inlet (Wright et al., 2012), and there is plasticity of electron donors and acceptors within the SUP05 (Anantharaman et al., 2013). Enigmatic groups such as SAR406, SAR86, and Arctic97B-4 also exhibit similar distributions to those of SAR324 and SUP05 and further highlight the links between deep-ocean and pelagic environments.

### PLUMES AS DISPERSAL VECTORS FOR VENT MICROBES, SYMBIONTS, AND LARVAE

Hydrothermal plumes entrain both focused and diffuse hydrothermal fluids and thus are potential dispersants of vent-associated organisms (Jackson et al., 2010). Indeed, vent-associated larvae have been observed in plumes (Mullineaux et al., 1995), where they are likely transported great distances to colonize vent sites (Mullineaux et al., 2010). As discussed previously (Dick and Tebo, 2010; Lesniewski et al., 2012), the microbial community of the Guaymas Basin hydrothermal plume is clearly distinct from seafloor communities (**Figure 4**). Despite this ecological boundary between hydrothermal seafloor and plume habitats, hydrothermal plumes could play a key role in the dispersal of seafloor hydrothermal organisms such as hydrothermal sediment and chimney-associated microbes. Consistent with this notion, deep sequencing of 16S rRNA genes from Guaymas Basin (Anantharaman et al., 2013) revealed the presence, albeit at low abundance, of candidate division bacteria that are common in seafloor hydrothermal environments (**Figure 4**). To our knowledge, this represents the first evidence of seafloor microbes in the rare portion of the plume microbial community at a chronic vent site [they have been detected previously in eruptive event plumes (Summit and Baross, 1998)], and suggests that plumes could indeed be an important mechanism of dispersal of deep-sea vent organisms.

Potential connections between free-living microbes in plumes and symbionts of hydrothermal vent animals have long been recognized, but views on the nature of this relationship are evolving. The similarity of free-living SUP05 in plumes to symbionts was noted with the original discovery of SUP05 (Sunamura et al., 2004). More recently, plume SUP05 were found to have and express genes for H_2_ oxidation (Anantharaman et al., 2013) that are highly similar to those of bathymodiolin mussel symbionts at deep-sea vents (Petersen et al., 2011), and symbiont-like methanotrophs were detected in the Guaymas Basin hydrothermal plume (Li et al., unpublished). The traditional view of these microbial symbionts is that they evolved from free-living ancestors to form a few distinct symbiont clades. However, recent analysis shows that symbionts are phylogenetically interspersed with free-living forms of the bacteria, suggesting numerous evolutionary transitions between symbiotic and free-living forms (Petersen et al., 2012). Furthermore, horizontal gene transfer has been implicated as a significant mechanism of metabolic evolution of the symbionts (Kleiner et al., 2012). Bathymodiolin symbionts are thought to be transmitted horizontally and acquired by each generation from the environment (Petersen et al., 2012), and the relatively low diversity of symbionts within animal populations suggests that the animals carefully select symbionts from the pool of diversity present within free-living communities. However, the mechanisms by which this selection takes place, the degree to which symbionts and free-living populations are genetically and ecologically distinct, and the time scales over which transitions between free-living and symbiotic lifestyles occur all remain intriguing frontiers for understanding the relationship between animal symbionts and free-living plume microorganisms.

### THE INFLUENCE OF BENTHIC AND PELAGIC HABITATS ON PLUME MICROBIAL COMMUNITIES

What controls the relative contribution of benthic versus pelagic microbes to plume microbial communities? Although the limited data on microbial communities in plumes provides few answers, there are some preliminary clues. Both physical and biological factors likely play a role in determining the balance of seafloor versus water column microbes in plumes. Physical factors include (i) the fluid flux and entrainment rate of the rising plume, (ii) the properties and habitability of the near-vent environment that could potentially be entrained, such as temperature and material properties (e.g., hard substrate versus easily transported sediments or biological material such as biofilms or dense animal communities), (iii) bathymetry of the seafloor surrounding the vent environment [e.g., bathymetric highs lead to rapid dilution and dispersal whereas restricted volumes such as Guaymas Basin or the Suiyo Seamount caldera tend to accumulate hydrothermal chemistry and biota (German and Von Damm, 2004)], and (iv) the bioenergetic potential of plume geochemistry (i.e., high concentration of energy-rich electron donors is more likely to support a seafloor-derived community in the plume). Biological factors that influence the balance of seafloor versus water column microbes likely hinge on the properties of the water mass in which venting takes place, including the cell density and the structure of the community with regard to metabolic potential. Denser microbial communities and those that hold a large portion of organisms able to utilize inorganic electron donors for lithotrophic growth will promote a greater water column contribution to plume microbial communities. Finally, the microbial growth response to temperature likely influences the degree to which seafloor microbes are metabolically active in hydrothermal plumes. Thermophiles or hyperthermophiles are unlikely to be metabolically active in cold hydrothermal plumes, whereas mesophiles or psychrophiles from lower-temperature seafloor habitats may indeed remain active at plume temperatures.

## METABOLIC AND FUNCTIONAL LINKAGES BETWEEN PLUMES AND OTHER MARINE HABITATS

Recent reports of genomic and metabolic plasticity within microbial groups such as SUP05 (Anantharaman et al., 2013) underscore the need to use caution when inferring microbial metabolism and function from 16S rRNA genes of plume populations, even at fine phylogenetic scales. On the other hand, the emergence of random shotgun metagenomic and metatranscriptomic approaches, driven by rapidly increasing throughput and decreasing costs of DNA sequencing, provides new opportunities to directly assess microbial metabolism and its effect on biogeochemistry in deep-sea hydrothermal plumes. Lesniewski et al. (2012) used a parallel metagenomic and metatranscriptomic approach to show that ammonium, methane, and sulfur are the primary energy sources in the Guaymas Basin hydrothermal plume. Genomes and transcriptomes of abundant microbial groups were subsequently reconstructed to reveal the genetic potential and expression of specific microbial populations (Baker et al., 2012; Anantharaman et al., 2013; Li et al., unpublished; Sheik et al., unpublished). Comparison of these large ‘omics datasets to those from other marine environments provides a view of the dynamics of both community-wide functions and specific microbial groups across distinct settings, and potentially provides powerful insights into the factors that govern microbial and ecosystem functions in the deep sea.

**FIGURE 5:**
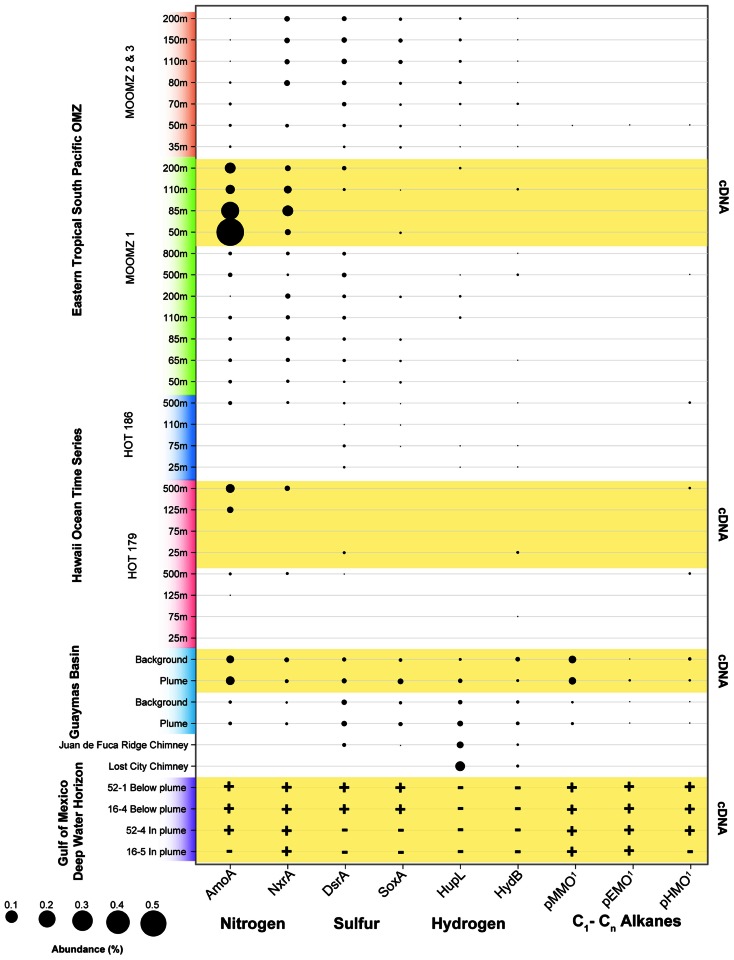
**Abundance of functional genes and transcripts for oxidation of selected nitrogen, sulfur, hydrogen, and hydrocarbon species in the following samples and studies: Eastern Tropical South Pacific OMZ (Stewart et al., 2012), Hawaii Ocean Time Series (DeLong et al., 2006), Guaymas Basin plumes and background (Lesniewski et al., 2012), and Deepwater Horizon oil spill (Goldstamp Gm00382, IMG TaxonID 2149837026)**. Metagenomic sequences from these studies were used as queries with BLASTX against databases containing the following sequences: AMO – ammonia monooxygenase subunit A (from both ammonia-oxidizing archaea and bacteria); Nxr – nitrite oxidoreductase subunit A; DsrA – dissimilatory sulfite reductase subunit A; SoxA – sulfur oxidation protein subunit A; HupL and HydB – group 1 membrane-bound Ni,Fe hydrogenase, large subunit; pMMO – particulate methane monooxygenase subunit A; pEMO – putative particulate ethane monooxygenase subunit A, includes sequences from Methylococcaceae bacterium species ET-HIRO (AB453962 and AB453963) and ET-SHO (AB453960 and AB453961), and environmental sequences (Redmond et al., 2010; Li et al., unpublished; SAR324_pHMO: putative C_2_–C_4_ alkane oxidizing monooxygenase subunit A, contains sequences from SAR324_J09 (Swan et al., 2011) and Guaymas Basin SAR324 (Li et al., unpublished; Sheik et al., unpublished). BLASTX bit scores >50 were considered positive matches. Abundance of sequence reads recruiting to each functional gene is shown as a percentage of total (putative) mRNA-containing cDNAs. rRNA were identified using Ribopicker [Version 0.4.3 (Schmieder et al., 2012)] with the comprehensive Ribopicker database “rrnadb” and removed from all datasets. The absence of data for samples simply indicates that it was not identified at the sequencing depth and does not necessarily imply the absence of genes/transcripts. Note that due to novelty of sequences, in some cases the pathway and substrate specificity are uncertain.

We analyzed the abundance of key functional genes in shotgun sequencing datasets from recent studies of the Guaymas Basin hydrothermal plume (Lesniewski et al., 2012), the Deepwater Horizon oil spill (Goldstamp Gm00382, IMG TaxonID 2149837026), the Eastern Tropical Pacific OMZ (Stewart et al., 2012), and the North Pacific subtropical gyre (DeLong et al., 2006; **Figure 5**). This data shows that functional genes for oxidation of nitrogen, sulfur, hydrogen, and hydrocarbons that are highly expressed in the Guaymas Basin hydrothermal plume are also widely present and expressed in these other disparate marine habitats (**Figure 5**). Genes for ammonia oxidation are abundant and highly expressed in nearly all datasets analyzed here except for surface waters of the ALOHA station, consistent with the widespread abundance of ammonia-oxidizing Archaea (Karner et al., 2001; Francis et al., 2005), specific populations of which are stimulated in the ammonium-rich hydrothermal plumes of Guaymas Basin (Baker et al., 2012). NXR (nitrite oxidoreductase) genes for nitrite oxidation (Lucker et al., 2010; Baker et al., 2013) were recovered from many of the datasets, especially OMZs. However, this result should be interpreted with caution given the novelty of NXR genes; it is difficult to distinguish the forms utilized by nitrite-oxidizing bacteria versus anaerobic ammonia oxidation (anammox) bacteria (Strous et al., 2006). Thus, we suspect that a significant fraction of NXR hits in the OMZ are from genes involved in anammox. Sulfur oxidation systems (*sox* and *dsr* genes) are most prevalent in the Guaymas Basin hydrothermal plume and in the OMZ cores, but they are also detectable in Guaymas Basin background and in some samples in the oxic water column at HOT (Hawaii Ocean Time Series). These sulfur oxidation genes are present in all metatranscriptomes of the Guaymas Basin, but only in a subset of those from the Gulf of Mexico, HOT, and ETSP OMZ. These results, especially expression of sulfur oxidation genes in oxic waters (HOT), are consistent with widespread cryptic geochemical cycling of sulfur (Canfield et al., 2010), perhaps in association with organic-rich particles (Karl et al., 1984; Wright et al., 2012). H_2_ oxidation genes are less widely distributed, but are most prevalent in ultramafic vent chimney samples (Brazelton et al., 2012) and in the Guaymas Basin hydrothermal plume, consistent with H_2_ being sourced primarily from hydrothermal fluids and being rapidly utilized in plumes (Kadko et al., 1990). However, H_2_ oxidation genes were also detected in some OMZ, ALOHA, and background Guaymas samples (Anantharaman et al., 2013), consistent with H_2_ production in association with organic-rich particles in the oxic water column also being a substantial source of H_2_ as predicted decades ago (Karl et al., 1984).

Utilization of methane and other hydrocarbons as an energy source represents another potential connection between hydrothermal plumes and other marine microbial habitats. The capability of naturally occurring marine microbes to consume hydrocarbons has recently been highlighted by the Deepwater Horizon disaster (Mason et al., 2012). While the connections need further investigation, natural sources of hydrocarbons in the deep sea (Jorgensen and Boetius, 2007) such as hydrocarbon seeps, hydrothermal vents, or *in situ* water column production (Karl et al., 2008) may prime deep ocean microbial communities for hydrocarbon degradation. Guaymas Basin hydrothermal fluids have unusually high concentrations of methane due to interaction with sediments that overlay the ridge. High methane concentrations are also found at the Endeavour Segment of the Juan de Fuca ridge (De Angelis et al., 1993), and ultramafic systems on the Mid-Atlantic Ridge (Charlou et al., 1998) and the Mid-Cayman Rise (German et al., 2010). The imprint of methane is clearly apparent in microbial communities of the Guaymas Basin hydrothermal plume; genes for pMMO are among the most abundant mRNAs represented in the metatranscriptome (Lesniewski et al., 2012). Recent work shows that there is extensive diversity of pMMOs and related Cu membrane monooxygenases at Guaymas, and that they affiliate phylogenetically with enzymes involved in C_2_–C_4_ alkane oxidation (Li et al., unpublished). We refer to these methane, ethane, and butane monooxygenases collectively as particulate hydrocarbon monooxygenases (pHMOs). pHMOs are also present in the metatranscriptome of the Deepwater Horizon oil spill (Mason et al., 2012; Li et al., unpublished) and in a few samples from station ALOHA (**Figure 5**). pHMOs were only detected at low levels in a few of the shotgun sequencing datasets, but they have been reported to be abundant in OMZs off Costa Rica (Tavormina et al., 2013).

## COMPARATIVE METATRANSCRIPTOMICS OF POPULATIONS IN PLUME AND BACKGROUND: SUP05 AND Thaumarchaeota

One of the remarkable conclusions emerging from comparison of different interfacial redox environments in the oceans is that the same microbial groups often dominate disparate environmental settings. For example, despite marked environmental differences between deep-sea hydrothermal plumes and OMZs (e.g., depth, temperature, pressure, nutrient availability, water mass history, quantity, and quality of DOC and POC), they share several of the most abundant microbial populations, including SUP05, SAR324, and *Thaumarchaea* (**Figure 4**). The nature of these two oxic/anoxic interfaces is quite different: in plumes, reduced chemicals are injected into an oxic water column, whereas in OMZs, reduced chemical species are produced through anaerobic microbial respiration of organic carbon. However, many of the microbial players appear to be the same, indicating that these organisms thrive at redox interfaces regardless of differences in other environmental parameters described above.

**FIGURE 6:**
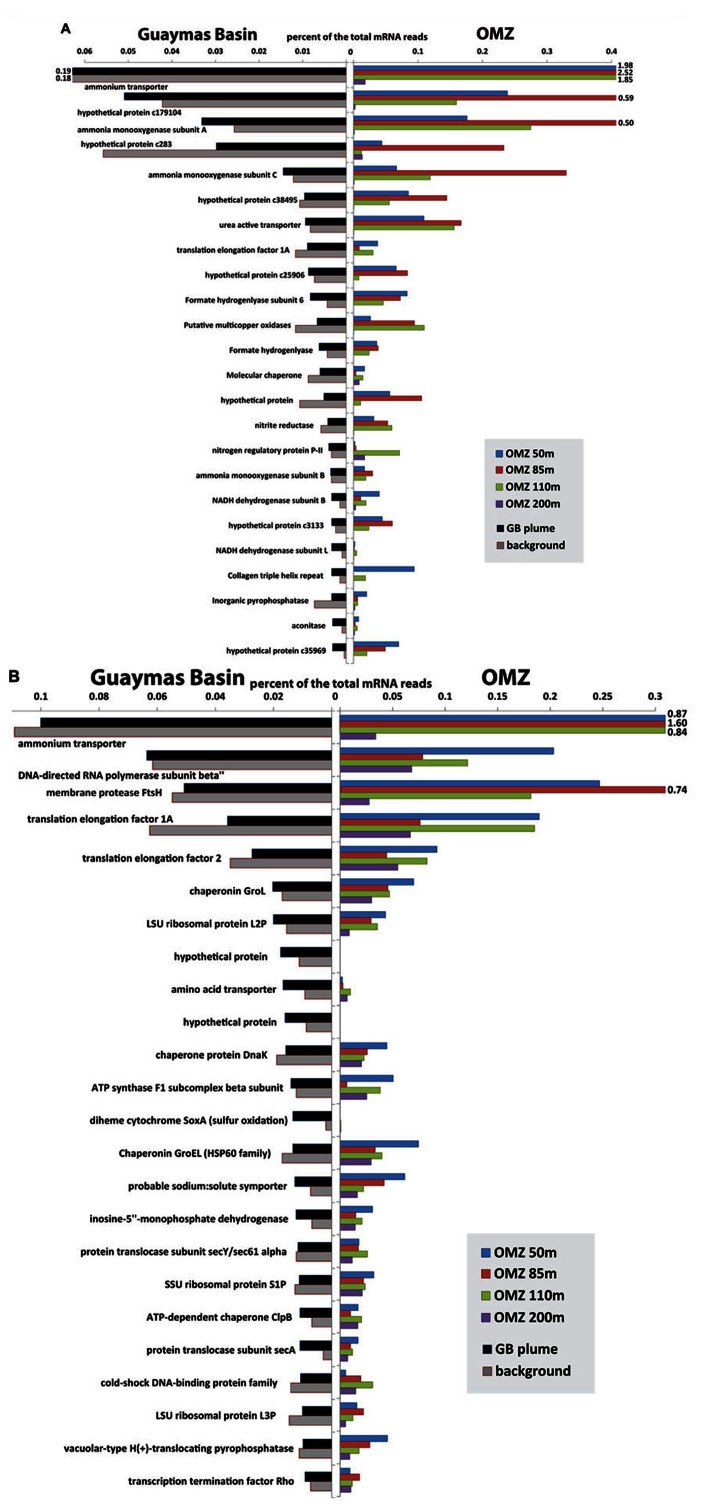
**Comparison of most abundant transcripts recruited to *Thaumarchaeota* (A) and SUP05 (B) between Guaymas Basin hydrothermal plume and Eastern Tropical South Pacific OMZ (Stewart et al., 2012)**. The mRNA reads (>70 bitscore) were recruited to the community genome assemblies of Thaumarchaea (Baker et al., 2012) and SUP05 (Anantharaman et al., 2013) from the Guaymas Basin with BLASTX. Bars that extend beyond the plot area are labeled.

Recent metatranscriptomic studies (Baker et al., 2012; Lesniewski et al., 2012; Stewart et al., 2012; Anantharaman et al., 2013) permit detailed views of the gene expression patterns of these microbial groups and their roles in biogeochemistry. Comparison of transcripts from OMZ and Guaymas Basin plume/background samples recruited to SUP05 and *Thaumarchaea* genomes shows that the overall patterns of transcript abundance are quite similar between the two environments (**Figure 6**). Indeed, the differences between samples *within* each environment (plume versus background at Guaymas, different depths of the OMZ) appear to be as significant if not greater than differences between environments. For *Thaumarchaea*, acquisition of ammonium and oxidation of ammonia dominate the metatranscriptome in both environments. Conserved hypothetical proteins of unknown function are similarly highly expressed in both environments, highlighting large gaps in our knowledge of what are likely critical functions for these organisms. In addition to obtaining ammonia directly from the environment, uptake of urea also appears to be an important source of ammonia for both populations (**Figure 6**). Utilization of urea has now been noted for *Thaumarchaeota* in diverse settings such as sponge symbionts (Hallam et al., 2006), soil (Tourna et al., 2011), polar waters (Alonso-Saez et al., 2012), and surface waters of the Gulf of Maine (Tully et al., 2012).

Like *Thaumarchaea*, transcript abundance profiles of SUP05 show striking similarities in the genes that are most highly expressed in plumes and OMZs (**Figure 6B**). Many of the highly expressed genes are involved in cell maintenance and growth (e.g., translation and DNA replication), which is expected in any environment. However, similarly high transcript abundance of other genes likely reflects common interactions between SUP05 and the two different environments. Ammonium transporters were the most highly expressed genes in all OMZ and plume samples except the 200 m depth of the OMZ. A predicted ABC (ATP-binding cassette) transporter for amino acids is expressed in all samples, but it appears to be most abundant in the deep sea.

SUP05 potentially plays an important role in linking the sulfur and nitrogen cycles by coupling the oxidation of reduced sulfur species to the reduction of nitrate or nitrite (Walsh et al., 2009; Canfield et al., 2010). Perhaps the most intriguing difference between plume and OMZ transcript profiles is that the *soxA* gene for sulfur oxidation is highly expressed in plumes but not in deep background waters or the OMZ. This suggests a difference in the form of sulfur used by SUP05 in plumes versus OMZs; abundant *soxA* transcripts point to thiosulfate oxidation in plumes, whereas free sulfide may be the preferred substrate in OMZs, as hypothesized by Walsh et al. (2009). Additional differences between populations of SUP05 include diversity in terms of electron donors and acceptors. SUP05 at the Saanich Inlet oxic/anoxic interface were described as anaerobes (Walsh et al., 2009), but SUP05-related symbionts appear to be aerobic (Kuwahara et al., 2007; Newton et al., 2007), and those in the Guaymas Basin plume are primarily aerobic (Anantharaman et al., 2013). Guaymas Basin SUP05 also have genes for H_2_ oxidation that do not appear to be present in other populations (Anantharaman et al., 2013).

## THREE BIG QUESTIONS FOR THE FUTURE OF DEEP-SEA HYDROTHERMAL PLUME MICROBIOLOGY

The recent explosion of new data on deep-sea microbial communities has revealed connections between deep-sea hydrothermal plumes and other marine habitats. These insights raise intriguing new questions regarding the roles of deep-sea hydrothermal systems in the chemistry and biology of the broader oceans. At the same time, revolutionary technological advances provide new opportunities to address these questions. One exciting future goal is to define how the interplay between microbiology and geochemistry in hydrothermal plumes extends to global-scale interactions between mid-ocean ridges and deep-sea microbiology. The global mid-ocean ridge hosts geochemically diverse hydrothermal systems. This geochemical diversity is tied to geological differences in the underlying host rock of hydrothermal systems, which shapes the quantity and types of metabolic energy available for chemoautotrophic growth in hydrothermal plumes (Amend et al., 2011). Indeed, vents show the highest variability in biodiversity of all marine systems (Zinger et al., 2011). Although microbial growth in plumes is predicted to be significant for carbon budgets in the deep oceans (McCollum, 2000), several first-order questions remain poorly constrained and need to be addressed.

### WHAT SHAPES THE STRUCTURE OF MICROBIAL COMMUNITIES IN DEEP-SEA HYDROTHERMAL PLUMES?

As we have seen, hydrothermal plume communities are composed of microbes from both seafloor and pelagic environments, and while data is scarce, the balance between these two sources appears to be variable and depends on both physical and biological factors. In addition to external contributions, chemoautotrophic growth *within* plumes likely contributes to the plume microbial community. Hence, the structure of communities in deep-sea hydrothermal plumes is shaped by both the geochemistry of the seafloor hydrothermal system (which shapes seafloor microbial communities and the energy available for growth in plumes) and the composition of communities in the surrounding water column. Microbial biogeography in the deep oceans is largely controlled by deep-sea circulation (Ghiglione et al., 2012) and hydrography (Galand et al., 2010), thus if the contributions from surrounding seawater are significant, then the geographic location of vents along the global deep conveyor belt of circulation could be an important determinant of plume community composition. Determining the relative importance of vent geochemistry and geography in shaping the community structure of plumes should be possible by tracking microbial diversity along local gradients of vent geochemistry in locations such as the Eastern Lau Spreading Center (Tivey et al., 2012). Expanded sampling and characterization of microbial communities in geographically widespread plumes in the broader deep sea will also be critical to evaluate biogeographic characteristics of deep-sea hydrothermal plumes.

### DO HYDROTHERMAL PLUMES INFLUENCE THE DIVERSITY OF DEEP-SEA MICROBIAL COMMUNITIES ON A GLOBAL SCALE?

So far we have focused on the contribution of background deep-sea microbes to plumes, but the interaction between communities in these two environments may in fact be bi-directional. Cell count data and thermodynamic modeling indicate that deep-sea hydrothermal plumes are productive environments compared to surrounding deep waters, and several studies have suggested that vents influence deep-sea microbial communities in their vicinity (Moyer et al., 1998; Takai et al., 2004). Active venting occurs in every ocean basin at over 1000 vent fields globally (Beaulieu et al., 2012), and surface-generated mesoscale eddies potentially transport hydrothermal plumes long distances (Adams et al., 2011). Although dispersion of plumes may be largely constrained by the ridge axis (Speer et al., 2003) or confined to a narrow corridor 10 km to either side of the ridge axis (German and Von Damm, 2004), deep-sea microbial communities are relatively stable (Ghiglione et al., 2012), so microbes exported from plumes could persist for long periods of time. Clearly there is potential, but to what extent does chemoautotrophic growth in plumes influence the broader deep oceans? The answer is poorly constrained, but given recent insights into cryptic chemoautotrophy throughout the oceans, the nature of potential influence takes on new meaning. Rather than being unique oases of chemoautotrophy in the oceans, perhaps vents should be viewed as one of many potential sources of electron donors that collectively maintain widespread chemoautotrophy in the oceans. Advances in thermodynamic-bioenergetic modeling of plumes on a global scale, incorporation of this data into ecological models, and evaluation of the results with experiments and observations provides great promise for addressing this question. Finally, even if the answer is that plumes do not influence broader deep-sea microbial communities, the value of deep-sea hydrothermal plumes as natural laboratories to examine the response of deep-sea microorganisms to diverse geochemical perturbations should not be ignored.

### HOW DOES MICROBIAL ACTIVITY IN DEEP-SEA HYDROTHERMAL PLUMES IMPACT CARBON BUDGETS OF THE DEEP OCEANS?

The deep oceans hold the largest reservoir of rapidly exchangeable inorganic carbon on the Earth’s surface. Microbial autotrophy potentially converts dissolved inorganic carbon into the organic phase within the biota. Subsequent grazing and viral predation transfer this carbon through the food chain or into the dissolved organic carbon pool. Although plume autotrophy likely has a negligible impact on the dissolved inorganic carbon inventories over global and short-term scales, its impact on organic carbon in the deep sea, especially on regional spatial scales, is potentially significant. In particular, the possibility that microbial autotrophy, heterotrophy, and/or lysis in hydrothermal plumes contribute to a significant conversion of inorganic matter to refractory organic matter, and thus sequestration of carbon as a component of the “microbial carbon pump” (Jiao et al., 2010), has not been explored in any detail. Improved measurements of plume microbial carbon fixation rates and studies of the fate of that carbon are required to evaluate this possibility. Another important but understudied aspect of microorganisms in deep-sea hydrothermal plumes is their role in modulating the flux of hydrocarbons from the seafloor through the water column and into the atmosphere (Orcutt et al., 2011). Future investigation on the inter relationship between plume microbial activity and hydrocarbon degradation will provide a better understanding of the impact of hydrothermal plume microbial activity on deep ocean carbon cycling.

Although this review has focused on hydrothermal plumes from high-temperature venting along the mid-ocean ridges, it should also be noted that low-temperature venting, including ridge-flank circulation, also likely contributes significantly to the processes described here. In fact, some estimates suggest that low-temperature ridge flank systems drive fluxes of fluid, heat, and solutes that are larger than those from high-temperature hydrothermal systems (Wheat and Mottl, 2000; Fisher and Harris, 2010). The microbial biogeochemistry of such systems is poorly known, but (McCarthy et al., 2011) showed that chemosynthesis by crustal microbial communities is a major source of dissolved organic carbon in ridge-flank and on-axis hydrothermal fluids sampled from the Juan de Fuca Ridge. Thus ridge-flank systems likely amplify the contributions of hydrothermal/subsurface circulation to the biology and geochemistry of the oceans and strengthen ecological and biogeographic connections between these systems.

## Conflict of Interest Statement

The authors declare that the research was conducted in the absence of any commercial or financial relationships that could be construed as a potential conflict of interest.
